# Supplementary material for: Patterns of Intron Gain and Loss in Fungi
Source: PLoS Biol. 2004 Nov 30;2(12):e422. doi: 10.1371/journal.pbio.0020422 (PMC532390; doi:10.1371/journal.pbio.0020422)
Supplement: Table S1 — Also available at http://genes.mit.edu/NielsenEtAl/. (4.3 MB ZIP). [file pbio.0020422.st001.zip › NielsenEtAl/html/1322.html]

AN2491.1.NCU04327.1.MG08122.1.FG02622.1


```
 CLUSTAL W (1.82) Multiple Sequence Alignments - Introns Inserted


Sequence 1: NCU04327.1	882 aa
Sequence 2: MG08122.1	865 aa
Sequence 3: FG02622.1	827 aa
Sequence 4: AN2491.1	875 aa
Alignment Length: 910 aa
Number Identitical Residues: 538 aa
Alignment Score (without introns) 23835


MG08122.1 	--~------MDSANRGLAAGSSNRKRARPD-DGTSSIAAASSP~-MPSSPPAFHMAHGAD
NCU04327.1	MS2SPLRDNPSSANRG-AVPRATRKRARNDHDGASSLPRASSP~VMPSSPPAFPVAHGAD
FG02622.1 	--~-----------------------------------MASSP~-MPSSPPEFNITHGVE
AN2491.1  	--~--------------MGNLNRRKRSREPDDDASSVVPPSSP1-PPSSPPMLPFDEDED
          	                        . :    ..:::   .***   ***** : . .. :

MG08122.1 	DNDDDIEED--AEIQDDIDEIDEMAEDD--VDLFRDGYERDYQQ-DENDRYEGIDIDDEG
NCU04327.1	E-DDDIEEDVAAEIEDDIDDLDELAEDD--VDLFREGFERDYRERDENDAYEGIDIDDD-
FG02622.1 	D-DDDIEEE--VMIQDDIDDIDEMAEDE--VDLFREGYENDYRD-LEDDHYGGEGIDDE-
AN2491.1  	EPDEEAELD--------IDDIEEMAEDEDGIDLFGDTFERDYRG-GKDDRYRGRYIDDD-
          	: *:: * :        **:::*:***:..:*** : :*.**:   ::* * *  ***: 

MG08122.1 	NYEGLDIAERRRLEAQLNRRDREVARRQRIPAAFLPGEDEDGDIDLTAQPRRRRHRYDED
NCU04327.1	EYEALDPAARRRLEAKLAQRDRQVR--MGMGTTYLPGDDDDGDIDLTNLERRRRIRYNED
FG02622.1 	QYEDMRIDQRQQVDKVLNRRDRAIQERRGMPQVWGALDEDGDDIDLSAQPRRRRHNYDEN
AN2491.1  	EQEEIDIGARRELEARLDRRDRELARRRRMPAAFLQ-DDEDGDIDLTAQPRRRRHAYDED
          	: * :    *:.::  * :*** :     :  .:   :::..****:   ****  *:*:

MG08122.1 	MDEDMDADIMAEELSLEALHDVKAASLTDWVSNPAVQRTIKREFKAFLTEYTDEHGSSVY
NCU04327.1	PDVDMDGDIMDSELPLEALMDVKAATLSEWISVPAVQKTIRREFKAFLTEYTDESGSSVY
FG02622.1 	PDDIMDTDIMSEELSLEALGDVKASSLTEWVSLPSVQRTIRREFKAFLTSYTDTSGSSVY
AN2491.1  	RDDIEMADDGLEELSLEELVDIKSSNITDWVTQPQVLRSIYREFKAFLTEFTDPTGSSVY
          	 *     *   .**.** * *:*::.:::*:: * * ::* ********.:**  *****

MG08122.1 	GNRIKTLGEVNAESLEVSYEHLAATKATLAYFLANAPTEILKLFDEVAMDVVLIHYPDYE
NCU04327.1	GNRIRTLGEINAESLEVSYEHLATAKAILAYFLANAPTEMLKLFDEVAMEVVLLHYPDYE
FG02622.1 	GSRIRTLGEVNAETLEVSYEHLSESKAILAYFLANAPAEMLKLFDEVAMDVVLLHYPDYE
AN2491.1  	GNKIKTLGEVNSASLEVSYTHLSSTKAVLGYFLANEPTEVLKVFDQVALDVTLFHYPQYH
          	*.:*:****:*: :***** **: :** *.***** *:*:**:**:**::*.*:***:*.

MG08122.1 	RIHSEIHVRIFDLPVHYTLRQLRQSHLNCLVRVSGVVTRRTGVFPQLKYVKVDCTKCGVT
NCU04327.1	RIHAEIHVRIFDLPIHYTLRQLRQSHLNCLVRVSGVVTRRTGVFPQLKYVKFDCTKCGVT
FG02622.1 	RIHSEIHVRIFDLPVHYTLRQLRQSHLNCLVRVSGVVTRRSGVFPQLKYVKFDCTKCGVT
AN2491.1  	DIHNEIHVRITDLPIVYTLRQLRQSHLNCLVRVSGVVTRRTGVFPQLKYVMFICQKCNMT
          	 ** ****** ***: ************************:********* . * **.:*

MG08122.1 	LGPFQQESNVEVKVTYCQNCQSRGPFTLNSEKTVYRNYQKLTLQESPGTVPAGRLPRQRD
NCU04327.1	LGPFQQESNVEVKISYCQSCQSRGPFTLNSEKTVYRNYQKLTLQESPGTVPAGRLPRHRE
FG02622.1 	LGPFQQESNVEVKITYCQSCQSRGPFTLNSEKTVYRNYQKLSLQESPGTVPAGRLPRSRE
AN2491.1  	LGPYQQESSSEVKVTMCQNCQSRGPFTVSSEKTVYRNYQKLTLQESPGSVPAGRLPRQRE
          	***:****. ***:: **.********:.************:******:******** *:

MG08122.1 	VVLLWDLIDKAKPGEEIEVTGTYRNNYDAQLNNRNGFPVFATILEANNIVKAHDQLAGFR
NCU04327.1	VILLWDLIDKAKPGEEIEVTGIYRNNYDAQLNNRNGFPVFATILEANNIVKSHDQLAGFR
FG02622.1 	VILLWDLIDKAKPGEEIEVTGIYRNNYDAQLNNRNGFPVFATILEANNVVKSHDQLAGFR
AN2491.1  	VILLADLIDSAKPGDEIEITGIYRNSYDAQLNNKNGFPVFATIIEANHVIKSHDQQAGFQ
          	*:** ****.****:***:** ***.*******:*********:***:::*:*** ***:

MG08122.1 	MTEEDEQEIRKLSKDPQIVDKIINSIAPSIYGHTDIKTAVALSLFGGVAKTAKGLHHVRG
NCU04327.1	MTEEDEHEIRRLSRDPHIVDKIINSVAPSIYGHTDIKTAVALSLFGGVAKQVG-AHHIRG
FG02622.1 	MTEEDEQNIRKLSRDPNIVDKIINSIAPSIYGHTDIKTAVALSLFGGVAKVTKGAHHLRG
AN2491.1  	LTEEDEREIRALSRDPDIVEKIIRSIAPSIYGHQDVKTAVALSLFGGVRKETQGKMSIRG
          	:*****::** **:**.**:***.*:******* *:************ * . .   :**

MG08122.1 	DINVLLLGDPGTAKSQVLKYAEKTAHRAVFATGQGASAVGLTASVRRDPLTSEWTLEGGA
NCU04327.1	DINVLLLGDPGTAKSQVLKYAEKTAHRAVFATGQGASAVGLTASVRRDPLTSEWTLEGGA
FG02622.1 	DINVLLLGDPGTAKSQVLKYAEKTAHRAVFATGQGASAVGLTASVRRDPLTSEWTLEGGA
AN2491.1  	DINVLLLGDPGTAKSQILKYVEKTAHRAVFATGQGASAVGLTASVRRDPLTSEWTLEGGA
          	****************:***.***************************************

MG08122.1 	LVLADKGTCLIDEFDKMNDQDRTSIHEAMEQQTISISKAGIVTTLQARCGIIAAANPIGG
NCU04327.1	LVLADKGTCLIDEFDKMNDQDRTSIHEAMEQQTISISKAGIVTTLQARCGIIAAANPIGG
FG02622.1 	LVLADRGTCLIDEFDKMNDQDRTSIHEAMEQQTISISKAGIVTTLQARCGVIAAANPIGG
AN2491.1  	LVLADRGTCLIDEFDKMNDQDRTSIHEAMEQQTISISKAGIVTTLQARCAVVAAANPIGG
          	*****:*******************************************.::********

MG08122.1 	RYNSTIPFSSNVELTEPILSRFDILCVVRDTVDPAEDERLARFIVGSHSRSHP-------
NCU04327.1	RYNSTIPFSANVELTEPILSRFDILCVVRDTVEPEEDERLARFIVGSHSRSHP-------
FG02622.1 	RYNSTAPFSSNVELTEPILSRFDILCVVRDTVEPEEDERLARFIVGSHSRSHP-------
AN2491.1  	RYNSSAPFSENVQLTEPILSRFDILCVVRDLVDPSEDERLASFVIESHHRANPSKPLRDE
          	****: *** **:***************** *:* ****** *:: ** *::*:..  ..

MG08122.1 	---TTMAQSQTADG--SMEVEHD-------SEQ-ADTQGS---ERKKEGEIPQDLLRKYI
NCU04327.1	---LMNNNTQDASGGDSMEVEHD-------TQAAAETQQTGEHGRKKEGEIPQELLRKYI
FG02622.1 	---LSQAEQG------SMEVEHD-------TQA--ETQGS---TRKPEGEIPQELLRKYI
AN2491.1  	HGNLINADGERIDE-EGYRIDDDGIRLPLRPDEIEARNAARDNDDEKEGEIPQELLRKYI
          	 ..    :    .  .. .::.*.   .  .:     : : .   : ******:******

MG08122.1 	KYARERCSPKLYHMDEDKVARLFADMRRESLATGAYPIT0VRHLEAIIRISEAFCRMRLS
NCU04327.1	LYARERCQPKLYHMDEDKVARLFADMRRESLATGAYPIT0VRHLEAIIRISEAFCRMRLS
FG02622.1 	LYAREHCSPKLYHIDEDKIARLFADMRRESIATGAIPIT0VRHLEAIIRISEAFCRMRLS
AN2491.1  	LYAREHCRPKLYQIDQDKVARLFADMRRESLATGAYPIT0VRHLEAIMRIAEAFCKMRLS
          	 ****:* ****::*:**:***********:**** *** *******:**:****:****

MG08122.1 	EYCTAQDIDRAIAVTVDSFVGSQKVSCKKALARAFAK~YTLARPGAGAGRRL---AGT--
NCU04327.1	EYCSAQDIDRAIAVTVESFVGSQKVSCKKALARAFAK~YTLNRPGTGNNGSG---SGTSQ
FG02622.1 	EYCAAQDIDRAIAVTVDSFVGSQKISCKKALARAFAK~YTLARPG-GQKKTG---ANS-Q
AN2491.1  	EYCSAQDIDRAIAVTVDSFIGSQKVSAKRALSRAFAK2YTLSRPKPQSKRRAGIPAPVSF
          	***:************:**:****:*.*:**:***** *** ** .      . .:  : 

MG08122.1 	GRGGRASAAASA--
NCU04327.1	GRRGGARRGVSAAA
FG02622.1 	GRRQTAAAS-----
AN2491.1  	ERRPQAAR------
          	 *   *
```
